# Production and response of a human prostatic cancer line to transforming growth factor-like molecules.

**DOI:** 10.1038/bjc.1990.333

**Published:** 1990-10

**Authors:** A. MacDonald, G. D. Chisholm, F. K. Habib

**Affiliations:** University Department of Surgery (WGH), Western General Hospital, Edinburgh, UK.

## Abstract

Serum-free media conditioned by the androgen insensitive human prostate cancer cell line DU145 showed immunological transforming growth factor-alpha (TGF alpha) activity, as well as competing activity in epidermal growth factor (EGF) radioreceptor assays (RRA). Furthermore, there were factors in the conditioned media which inhibited and stimulated DNA synthesis by DU145 cells in a dose-dependent fashion. Fractionation of the concentrated conditioned media by reverse-phase high performance liquid chromatography revealed several peaks containing EGF-like competitive activity only one of which demonstrated TGF alpha activity. However, none of the peaks corresponded to immunoreactive EGF. Measurement of EGF receptors on DU145 cells by competition and saturation analysis revealed high levels of receptors (mean +/- s.d. = 2.5 +/- 1 x 10(5) surface receptors per cell) which were of high affinity (Kd +/- s.d. = 1.0 +/- 0.5 nmol l-1). Although DU145 cells express high levels of EGF receptors, DNA synthesis was only minimally affected by exogenous EGF and TGF alpha.


Br. J. Cancer (1990), 62, 579-584 © Macmillan Press Ltd., 1990

### Production and response of a human prostatic cancer line to transforming growth factor-like molecules

A. MacDonald, G.D. Chisholm & F.K. Habib

*University Department of Surgery (WGH), Western General Hospital, Edinburgh EH4 2XU, UK.*

**Summary** Serum-free media conditioned by the androgen insensitive human prostate cancer cell line DU145 showed immunological transforming growth factor-α (TGFα) activity, as well as competing activity in epidermal growth factor (EGF) radioreceptor assays (RRA). Furthermore, there were factors in the conditioned media which inhibited and stimulated DNA synthesis by DU145 cells in a dose-dependent fashion. Fractionation of the concentrated conditioned media by reverse-phase high performance liquid chromatography revealed several peaks containing EGF-like competitive activity only one of which demonstrated TGFα activity. However, none of the peaks corresponded to immunoreactive EGF. Measurement of EGF receptors on DU145 cells by competition and saturation analysis revealed high levels of receptors (mean ± s.d. = $2.5 \pm 1 \times 10^5$ surface receptors per cell) which were of high affinity (Kd ± s.d. = $1.0 \pm 0.5$ nmol $l^{-1}$). Although DU145 cells express high levels of EGF receptors, DNA synthesis was only minimally affected by exogenous EGF and TGFα.

The secretion of growth factors by some transformed cells is thought to enable these cells to proliferate in low serum concentrations as well as reducing the dependency upon exogenous growth factors. One mechanism of transformation is thought to involve cells being able to produce and respond to growth factors (autocrine secretion), thus conferring a growth advantage over other cells (Sporn & Todaro, 1980).

Transforming growth factors (TGFs) and other growth factors have been implicated in the autocrine and/or paracrine growth of tumour cells (Sporn & Roberts, 1985). A lot of interest has arisen in TGFs, as they are a family of peptides which confer upon non-transformed cells several properties associated with the transformed phenotype, such as anchorage-independent growth in semisolid medium (Brown & Blakely, 1984). TGFs are also unique in that their expression is elevated in transformed cells (Marquardt *et al.*, 1983).

One class of TGFs is the TGFαs. They are potent mitogens for a number of cell types and bind to and interact with the epidermal growth factor (EGF) receptor (Massague, 1983). TGFαs have significant sequence and structural homology to EGF and show a variety of biological actions similar to EGF. Their biological activities when assayed in cell culture systems are almost identical (Bascom *et al.*, 1989), although in some cases TGFs are more potent than EGF (Stern *et al.*, 1985; Ibbotson *et al.*, 1986).

TGFαs have been identified and purified from various sources. Biologically active TGFαs have been demonstrated in crude or partially purified extracts prepared from rodent and human mammary tumours and in the conditioned medium (CM) obtained from several human cell lines (Zwiebel *et al.*, 1982; Brown & Blakely, 1984; Moses, 1984; Salomon *et al.*, 1984, 1986; Dickson *et al.*, 1986; Perroteau *et al.*, 1986). In addition TGFα has been shown to be produced by the hormone responsive LNCaP prostate cell line (Wilding *et al.*, 1989). However, it is not yet clear whether the hormone independent prostate cancer cells secrete their own TGFα as part of an autocrine regulatory mechanism. In the present study we demonstrate that the hormone insensitive human prostate cancer cell line DU145 produces bioactive EGF-like molecules, which are immunologically related to TGFα. We also examine the relationship between TGFα production and the EGF receptor and the response of the DU145 cells to the secretory products in the conditioned media as well as to exogenous TGFα and EGF.

Correspondence: F.K. Habib.
Received 8 February 1990; and in revised form 14 May 1990.

### Materials and methods

### *Hormones and growth factors*

EGF from mouse submaxillary gland (receptor and tissue culture grade), was purchased from Collaborative Research (Universal Biologicals, St Ann's, London). Human EGF (or urogastrone) was kindly donated by H. Gregory (ICI, Macclesfield, UK). Rat TGF-1 (rTGF-1) was purchased from Peninsula Laboratories Europe Ltd (St Helens, Merseyside, UK) as part of a radioimmunoassay kit.

### *Cell culture*

The prostatic human carcinoma cell lines DU145 was used in all experiments. This cell line is an androgen independent adenocarcinoma, originally isolated from a brain metastasis (Stone *et al.*, 1978) as was obtained from Dr D. Mickey (Department of Urology, University of North Carolina, USA).

The cells were maintained at 37°C in a humidified atmosphere of 95% air and 5% $CO_2$ in 75 $cm^2$ tissue culture flasks (Corning, Staffordshire, UK) in serum free media. A serum free DU145 cell line was developed for the purposes of this study by repeated subculture at high density in RPMI 1640 (Flow Laboratories, Irvine, UK), supplemented with serum free constituents: transferrin (10 mg $l^{-1}$), insulin (10 mg $l^{-1}$), hydrocortisone (1 mg $l^{-1}$), phosphoethanolamine (50 μg $l^{-1}$), 3,3',5-tri-iodo-thyronine (0.04 nmol $l^{-1}$; Sigma, Poole, Dorset, UK), trace element mix (1 mg $l^{-1}$; Gibco, Irvine, Scotland), L-glutamine (1%; Flow Laboratories) and penicillin/streptomycin (0.8%; Gibco).

### *Preparation of conditioned medium*

Cells were grown to confluence in 75 $cm^2$ tissue culture flasks in serum free medium (SFM), the medium was changed once, discarded and the cells were grown for a further 48 h in 20 ml of SFM. The conditioned media (CM) was clarified by centrifugation (3,000 r.p.m. for 15 min) and filtered through a 0.2 μm filter (Gelman Sciences Inc., Ann Arbor, Michigan, USA). At this stage CM was stored at −20°C until further use. Pooled CM from 50 flasks (1 litre) were dialysed against ammonium acetate (50 mmol $l^{-1}$) in Spectrapor 3 dialysis tubing at 4°C (mol. wt cut-off point 3,500; Spectrapor, Pierce-Warriner, Chester, UK), after addition of phenylmethylsulphonyl fluoride (0.3 mmol $l^{-1}$; Sigma). The dialysed CM was subsequently lyophilised to dryness. The lyophilised material was reconstituted in 5 ml of PBS (0.02 M $Na_2HPO_4.2H_2$,$NaH_2PO_4.2H_2O$, 0.9% NaCl, pH 7.4) and clarified by centrifugation at 10,000 $g$ for 30 min.

Protein concentration was determined by the method of Bradford (1976) using bovine serum albumin as standard.

### $^3$H-Thymidine incorporation

Subconfluent cells were rinsed in Dulbecco 'A' phosphate buffered saline (PBS; Oxoid Ltd, UK), and once with 0.25% trypsin and 0.2% EDTA (Gibco). The cells were incubated at 37°C for 5 min and subsequently resuspended in serum free medium (SFM). Cell counts were determined in triplicate dishes set up alongside the experimental dishes using a haemocytometer chamber. Ten thousand cells were plated overnight in SFM supplemented with 0.5% fetal calf serum (FCS; Gibco) to assist plating, in 96-well plates (Cell-Cult). The plating media was then replaced by SFM with either EGF, TGFα or an aliquot of the CM for up to 72 h. After the incubation period, methyl-$^3$H-thymidine (37 kBq per well, specific activity 75 GBq mmol$^{-1}$; Amersham International plc) was added in RPMI for at least 4 h. The medium in each well was aspirated, resuspended before the addition of 10% ice-cold trichloroacetic acid (TCA). The cells were harvested 2 h later on to filter mats (Skatron Combi Cell harvester) by washing the well three times in water and then drying the filter mats at 60°C for 30 min. Each disc of filter paper containing the precipitable material was then counted in scintillation fluid.

The growth factors EGF and TGFα were tested for an effect on DNA synthesis of DU145 cells, over the range 0.01–10 nmol l$^{-1}$. CM was also tested over the concentration range 0.01–1,000 µg lyophilised CM ml$^{-1}$.

### EGF radioreceptor assay

Binding assays were carried out on DU145 monolayers, in 24-well plates (Cell-Cult) at a cell density of $2 \times 10^5$ cells per well. Before addition of the binding medium, the SFM from each well was aspirated and the cells and binding media were cooled on ice to inhibit receptor internalisation. The cell monolayers were then gently washed with Dulbecco 'A' PBS and binding was initiated upon the addition of the appropriate concentration of $^{125}$I-EGF (Amersham; specific activity 4 GBq µg$^{-1}$), in 0.5 ml of 0.02 mol l$^{-1}$ PBS (0.02 mol $Na_2HPO_4.2H_2O$ l$^{-1}$, 0.02 mol $NaH_2PO_4.2H_2O$ l$^{-1}$, 0.9% NaCl, 1 g l$^{-1}$ BSA) pH 7.4. Non-specific binding was determined in the presence of 100-fold excess unlabelled EGF or CM in RPMI 1640. All binding studies were carried out for 3 h at 4°C. Unbound $^{125}$I-EGF was separated from cell bound $^{125}$I-EGF by washing the cells 3 × in Dulbecco 'A' PBS. The cells were subsequently solubilised with 1 ml 0.5 N NaOH, for 15 min and transferred to plastic tubes for counting in a gamma counter. Specfic binding was calculated as the difference between total binding and non-specific binding. Cell numbers were determined from control wells (triplicate wells) in which RPMI 1640 was added, without EGF.

### Saturation and competition analysis

Evaluation of binding parameters were obtained by saturation analysis over the range 0.05–8 nmol l$^{-1}$ $^{125}$I-EGF with or without excess unlabelled EGF. The dissociation constant (Kd) and the number of binding sites ($R_T$) were also measured by competition analysis with increasing concentrations of EGF (0.01–200 nmol l$^{-1}$) and a constant amount of $^{125}$I-EGF (2 nmol l$^{-1}$).

### Radioreceptor competition assay (RRA)

DU145 cell monolayers were used as the source of EGF receptors in the EGF-RRA, for analysis of competitive activity in the CM. The amount of EGF equivalent units in samples of CM was calculated by comparison with the competition curves derived from unlabelled hEGF, with $^{125}$I-EGF competing for EGF binding sites on DU145 cells. In this assay the sensitivity for unlabelled mEGF, hEGF or TGFα is approximately 0.12 ng per assay with 1 ng of $^{125}$I-EGF and 50% competition occurring at 0.6 ng per assay.

### Reverse-phase high performance liquid chromatography

The lyophilisate of CM stored at −70°C was reconstituted in 5 ml of PBS. Trifluro acetic acid (TFA; 0.1%) was added to an aliquot (10 mg) of the CM, vortexed and spun at 15,000 r.p.m. for 10 min. The soluble material was loaded on to a Bio-Rad RP 304 $C_4$ column (250 mm × 10 mm), with a slurry packed guard column of the same material. The column was equilibrated with 95% Buffer A (0.2 µm filtered double distilled water (ddw), 0.1% TFA) and 5% Buffer B (20% ddw, 80% acetonitrile (MeCN), 0.085% TFA), and the sample eluted with a linear gradient of 5–80% Buffer B, with a flow rate of 0.5 ml min$^{-1}$ at 22°C. The absorbence of each fraction was read at 280 nm with 1 ml fractions collected. Acetonitrile was blown off each fraction under a stream of air, over 24 h, and the remaining sample frozen to −70°C, lyophilised to dryness and reconstituted in 0.5 ml of 0.02 M PBS with 1 g l$^{-1}$ BSA, pH 7.4. Every third fraction was analysed for competitive activity in an EGF-RRA as previously described and for hEGF and rTGF-1 immunological activity by RIA.

### Radioimmunoassays for TGFα and hEGF

The amount of immunoreactive TGFα and hEGF in samples of CM and in the rHPLC fractions were determined using a liquid-phase competitive RIA. Detection of TGFα was performed using a commerical kit (Peninsula Laboratories Inc.) with rTGF I as radioiodinated tracer and reference standard. Antisera to the rTGF I were raised in rabbits and purchased from Peninsula Laboratories Europe Ltd. The rabbit anti-rat TGF I antiserum recognises both mouse and human TGFα but does not cross react with either mouse or human EGF. Half-maximal inhibition of binding of the $^{125}$I-peptide to the antibody occurred at 100 pg per tube. EGF-life activity was determined from the competition of hEGF for $^{125}$I-EGF binding to anti hEGF serum. Briefly antiserum raised in rabbits to purified hEGF (kindly provided by Dr H. Gregory, ICI Macclesfield, UK) was incubated at 4°C with $^{125}$I-EGF (10,000 c.p.m. per assay tube) and hEGF standards (0–20 ng in triplicate) or CM samples. After 3 days donkey anti-rabbit polyclonal IgG (Scottish Antibody Production Unit, Carluke, UK) was added for 24 h at 4°C. The assay tubes were then centrifuged at 3,000 r.p.m. for 30 min. The supernatant was aspirated and the resultant radiolabel in the pellets was determined by counting in a gamma counter. Half-maximal competition was observed with 1–2 ng native hEGF.

### Data analysis

The computer analysis employed for competition and saturation analysis was the weighted, non-linear least-squares curve fitting program LIGAND (DeLean et al., 1978; Munson & Rodbard, 1980). Data were analysed according to a model for one or two binding sites. A model for two binding sites is retained only when it fits the data significantly better ($P < 0.05$ partial F test) than a model for a single binding site.

Statistical significance in the growth experiments was determined using a two-tailed Student's $t$ test for comparison of means.

### Results

### Production of EGF-like competing factors

To examine for the presence of specific EGF-like molecules in the conditioned media from DU145 cells, increasing concentrations of CM were tested for their ability to compete with $^{125}$I-EGF for binding to DU145 cells (Figure 1). Growth factors present in the CM inhibited $^{125}$I-EGF in a dose

**Figure 1** Production of competitive EGF RRA activity from medium conditioned by DU145 cells. Increasing concentrations of DU145 conditioned media (CM) were assayed for EGF RRA competitive activity. $^{125}$I-EGF 2 nmol l$^{-1}$ was incubated with or without increasing concentrations of CM for 3 h at 4°C. Unbound, $^{125}$I-EGF was separated from cell bound $^{125}$I-EGF by washing the cells 3 × in Dulbecco 'A' PBS. The cells were subsequently solubilised with 1 ml 0.5 N NaOH and the radioactivity remaining measured in a gamma counter. The amount of EGF equivalent units in the CM was calculated by comparison to the competition curves produced by unlabelled EGF with $^{125}$I-EGF (inset). The experiment was repeated twice, and each data point represents the mean ± s.d. ($n = 6$).

dependent fashion. By comparison to the competition curves produced by unlabelled hEGF with $^{125}$I-EGF (inset of Figure 1), the amount of EGF eqivalent units in 1 litre of media conditioned by DU145 cells was calculated to be approximately 30 ng.

### *Production of immunoreactive EGF and TGFα*

Equivalent amounts of concentrated CM were assessed for immunoreactivity using rTGF-1 and hEGF radioimmunoassays. The relative amounts of immunoreactive species produced by DU145 cells were compared with the levels of biologically active EGF-like molecules that competed with $^{125}$I-EGF for binding to DU145 monolayers (Table I). Approximately 20.3 ng l$^{-1}$ of immunoreactive TGFα was produced by DU145 cells compared with 30 ng l$^{-1}$ of EGF equivalent molecules. As no EGF was detected when CM was assayed for hEGF by RIA, then the remaining 32% of bioactive EGF-like activity, not related immunlogically to TGFα could not be attributed to the production of EGF.

### *Effect of conditioned media on $^{3}$H-thymidine incorporation*

Increasing concentrations of pooled and concentrated CM produced from DU145 cells were added to DU145 cell monolayers for up to 72 h and subsequently tested for their effect on DNA synthesis. Table II shows that CM at 30 μg ml$^{-1}$ induced a stimulation in thymidine incorporation at all time points and for all but 72 h, this stimulation was significant ($P < 0.05$). However, the largest increase achieved was after 24 h (Table II) and subsequently all experiments were carried out at this time point. The results demonstrating the optimal effect on DNA synthesis at 24 h are shown in Figure 2. Factors in the conditioned media had a biphasic effect on DNA synthesis, with concentrations from 0.01 to 1 μg ml$^{-1}$

**Table I** The presence of EGF receptor competing activity and immunoreactive TGFα in the DU145 cells conditioned media

| *Assay* | *Amount (ng l$^{-1}$ CM)* |
|---|---|
| RIA | |
| hEGF | 0.0 ± 0.0 |
| rTGF-1 | 20.3 ± 2.4 |
| RRA | 30.0 ± 10.1 |

**Table II** Effect of DU145 CM (30 μg ml$^{-1}$) on thymidine incorporation at different time points

| *Time (hours)* | *$^{3}$H-Thymidine incorporation (% of control ± s.d.)* |
|---|---|
| 8 | 136 ± 12 ($P < 0.05$) |
| 24 | 146 ± 28 ($P < 0.001$) |
| 48 | 135 ± 14 ($P < 0.05$) |
| 72 | 139 ± 40 ($P < 0.05$) |

DU145 cells (1.0 × 10$^{4}$ cells per well) were seeded in 96-well plates and 30 μg ml$^{-1}$ of CM was added for 8 ($n = 6$), 24 ($n = 12$), 48 ($n = 6$) and 72 h ($n = 6$) and $^{3}$H-thymidine for a further 4 h. Thymidine incorporation into DNA was measured by precipitating the cellular material with 10% ice cold TCA. The data is expressed as the percentage of $^{3}$H-thymidine incorporated relative to the untreated SFM control ± s.d.

**Figure 2** Dose-response effect of DU145 CM on $^{3}$H-thymidine incorporation in DU145 cells. DU145 cells (1.0 × 10$^{4}$ cells per well) were seeded in 96-well plates and increasing concentrations of CM (0.01–1,000 μg ml$^{-1}$) added for 24 h and $^{3}$H-thymidine for a further 4 h (37 Bq per well). After this time the amount of $^{3}$H-thymidine incorporated into DNA was measured by precipitating the cellular material with 10% ice-cold TCA, added for 2 h. The experiment was conducted over the concentration range 0.01–1 μg ml$^{-1}$ ($n = 6$) and range 3–1,000 μg ml$^{-1}$ ($n = 12$). The data are expressed as the percentage of $^{3}$H-thymidine incorporated relative to the untreated SFM control ± s.d.

having a marked inhibitory effect on incorporation of $^{3}$H-thymidine (50% inhibition relative to the untreated SFM control where the control is expressed as 100%; $P < 0.001$). However, this inhibitory effect on DNA synthesis was overcome by increasing the concentrations of CM: with a concentration of 30 μg ml$^{-1}$, $^{3}$H-thymidine incorporation was increased by 46 ± 28% ($P < 0.05$) relative to the SFM control. The marked decline in $^{3}$H-thymidine incorporation with higher concentrations of CM was possibly due to dilution of nutrients in the media.

### *rHPLC profile of conditioned media*

Media conditioned by DU145 cells was chromatographed using rHPLC and the fractions examined for immunological rTGF 1, hEGF activity and EGF radioreceptor competitive activity (Figure 3). Associated with the three major 280 nm absorbence peaks (1 Au) in the middle of the gradient was a minor peak of EGF radioreceptor competitive activity (20%

**Figure 3** Reverse-phase HPLC of concentrated DU145 CM. Trifluroacetic acid (0.1%) was added to concentrated CM (10 mg). The soluble material was loaded on to a Bio-rad RP 304C4 column (250 mm × 10 mm) and subjected to rHPLC; 200 μl aliquots of every third fraction was assayed for competitive activity in an EGF RRA (- O -), and 100 μl aliquots assayed for immunological hEGF and rTGF-1 activity by RIA (- ■ -). Protein was determined by absorbence at 280 nm (-). The linear MeCN gradient is marked by the continuous line.

**Figure 4** Dose-response effect of EGF and TGFα on DNA synthesis in DU145 cells. Cells were seeded in 96-well plates (1 × 10$^4$ cell per well), and after plating EGF or TGFα (0.01-10 nmol l$^{-1}$) was added for 24 h. $^3$H-Thymidine (37 Bq per well) was then added for 4 h, the cells were trypinised and 10% ice-cold TCA added for 2 h. The cells were then harvested on to filter mats, dried and counted in scintillation fluid. Each data point represents the mean ± s.d. ($n$ = 24) of three separate experiments and the data are normalised relative to the untreated SFM control (100%).

competition). There were also two other major peaks of competitive activity eluted at the beginning and end of the run (40-50% competition). The only peak of immunoreactive rTGF 1 (fractions 12-19), which eluted with 25-35% acetonitrile also demonstrated EGF-like competitive activity (40% competition). All the fractions were tested for immunoreactive hEGF, but hEGF was not detected in any of the rHPLC fractions.

### *The effect of exogenous EGF and TGFα on DNA synthesis*

The effect of exogenous EGF and TGFα on DNA synthesis was measured by the incorporation of radiolabelled thymidine (Figure 4). Preliminary studies were carried out to examine the effect of EGF on DNA synthesis over a period of up to 72 h. Maximum stimulation was achieved after 24 h (data not shown) and subsequently all experiments were therefore performed after 24 h. Both EGF and TGFα stimulated $^3$H-thymidine incorporation into DU145 cells in a dose-dependent manner. In the presence of 1 mmol EGF l$^{-1}$ thymidine incorporation was increased by 26 ± 13% ($P<$ 0.001) whereas TGFα maximally stimulated thymidine incorporation at a concentration of 0.03 nmol l$^{-1}$ by 17 ± 10% ($P<0.05$). Similar results were obtained using cell numbers as an index of proliferation (data not shown).

### *Saturation and competition studies*

EGF receptor binding parameters (Kd and Bmax) were estimated by competition and saturation analysis (Figure 5). Competition studies were carried out with increasing concentrations of unlabelled EGF and a constant amount of labelled ligand (2 nmol l$^{-1}$). Unlabelled EGF effectively competed for $^{125}$I-EGF binding, with a dissociation constant (Kd) of 0.6 ± 0.5 nmol l$^{-1}$. The results fitted significantly to a one site receptor model as can be seen by the sigmoidal shape of the curve ($P<0.05$) with (3 ± 1) × 10$^5$ receptor binding sites per cell. Increasing concentrations of $^{125}$I-EGF saturated the receptor binding sites (Figure 5). The results fitted significantly to a one class of binding site ($P<0.05$) with an estimated Kd value of 1 ± 0.5 nmol l$^{-1}$ and a Bmax of (2 ± 0.8) × 10$^5$ per cell. Overall results from competition and saturation analysis using the LIGAND curve fitting program gave a Kd value of 1 ± 0.6 nmol l$^{-1}$ with (2.5 ± 1.2) × 10$^5$ receptor binding sites per cell.

Binding parameters were evaluated only by competition and saturation analysis, due to the difficulty in estimating the lowest ligand concentrations, which are unevenly 'weighted' in Scatchard analysis (Bennett & Yamamura, 1985).

### Discussion

The results of this study demonstrate that the human prostatic cancer cell line DU145 produces molecules with TGFα-like immunoreactivity and competitive activity in EGF radioreceptor assays; this suggests that this cell line may be involved in a TGFα-mediated autocrine loop. This is further substantiated by the presence of high affinity receptors for EGF on the cell surface. The DU145 cells in our experiments also responded to exogenous EGF and TGFα by showing an increase in $^3$H-thymidine in corporation. However, this response was minimal which suggests that these cells may be producing sufficient quantities of endogenous TGFαs such that they are generally insensitive to proliferation by exogenous growth factors. The presence or lack of minimal proliferative response to exogenous growth factors has also been observed with other cell lines producing TGFαs (Salomon *et al.*, 1987; Verbeck *et al.*, 1988; Valverius *et al.*, 1989; van Zoelen *et al.*, 1987; Coffey *et al.*, 1986), although receptors are in most cases expressed. However, the presence

**Figure 5** Competition and saturation analysis. Confluent DU145 monolayers were incubated with $^{125}$I-EGF (2 nmol l$^{-1}$) and increasing concentrations of unlabelled EGF (0.01–100 nmol l$^{-1}$) for 3 h at 4°C or with increasing doses of $^{125}$I-EGF (0.02–8 nmol l$^{-1}$) and a constant amount of unlabelled EGF. The monolayers were subsequently washed 3 × with Dulbecco 'A' PBS, and the remaining radioactivity measured. The affinity constant and the number of EGF binding sites were evaluated using the binding program LIGAND. Three separate experiments were carried out for each analysis.

of receptors does not necessarily mean that a cell responds to a factor. In fact the loss of an EGF requirement after malignant transformation is well described (Cherington *et al.*, 1979). Wollenberg *et al.* (1989) noted that rat hepatocytes expressed receptors for EGF but that the high affinity receptors were not involved in the proliferative response.

Media conditioned by DU145 cells had a significant stimulatory and inhibitory effect on DNA synthesis, which was dose responsive. Although other workers had examined the relationship between immortal human prostate cell lines and exogenous growth factors (Connolly & Rose, 1989; Wilding *et al.*, 1989), this is the first that an effect by CM was reported in human prostate cancer cells. The biphasic effect canñot, at this stage, be attributed solely to growth factor production, nonetheless it is interesting to note that this cell line is endogenously producing stimulatory and inhibitory 'factors' which are influencing the growth of these cells and this warrants further investigation.

Using an immune-specific RIA, we demonstrated the presence of TGFα in medium conditioned by this cell line. However, stoichiometrically the level of immunologically related TGFα was not equivalent to the amount of EGF-like competitive activity which suggests that this cell line is producing forms of EGF related polypeptides distinct immunologically from TGFα and hEGF. This was further verified using a HPLC as we identified several molecular forms of EGF receptor binding polypeptides in medium conditioned by DU145 cells. Only one of three peaks demonstrated TGFα immunoreactivity and none were related to hEGF. It is interesting to note that many other types of cancer cells produce several molecular forms related to TGFα (Dickson *et al.*, 1986; Stromberg *et al.*, 1986; Salomon *et al.*, 1987). The TGFα related molecular species produced by the DU145 prostate cell line may comprise the precursor form of TGFα or intermediate forms; further studies are required to distinguish between these possibilities. Nevertheless, this raises the possibility that it may be possible for a cell to make subtle changes in response by producing different molecular forms of TGFα. We conclude, therefore, that DU145 by producing its own growth factors has little or no need for exogenous EGF and TGFα; the levels of growth factors produced may be auto-stimulatory, although this remains to be determined.

The authors wish to thank Dr J. Sharkey for his assistance with data analysis and Dr M. Collins for comments on the manuscript.

### References

BASCOM, C.C., SIPES, N.J., COFFEY, R.J. & MOSES, H.L. (1989). Regulation of epithelial cell proliferation by transforming growth factors. *J. Cell. Biochem.*, **39**, 25.

BENNETT, J.P. Jr & YAMAMURA, H.I. (1985). Neurotransmitter, hormone, or drug receptor binding methods. In *Neurotransmitter Receptor Binding*, 2nd edn, p. 61. Raven Press: New York.

BRADFORD, M.M. (1976). A rapid and sensitive method for the quantitation of microgram amounts of protein utilising the principle of protein dye binding. *Anal. Chem.*, **72**, 248.

BROWN, K.D. & BLAKELY, D.M. (1984). Transforming growth factors: sources, properties and possible roles in normal and malignant cell growth control. *Biochem. Soc. Trans.*, **12**, 168.

CHERINGTON, P.V., SMITH, P.L. & PARDEE, A.B. (1979). Loss of epidermal growth factor requirement and malignant transformation. *Proc. Natl Acad. Sci. USA*, **76**, 3937.

COFFEY, R.J. Jr., SHIPLEY, G.D. & MOSES, H.L. (1986). Production of transforming growth factors by human colon cancer lines. *Cancer Res.*, **46**, 1164.

CONNOLLY, J.M. & ROSE, D.P. (1989). Secretion of epidermal growth factor related polypeptides by the DU145 human prostate cancer cell line. *Prostate*, **15**, 177.

DELEAN, A., MUNSON, P.J. & RODHARD, D. (1978). Simultaneous analysis of families of sigmoidal curves: application of bioassay, radioligand assay, and physiological dose-response curves. *Am. J. Physiol.*, **235**, E97.

DICKSON, R.B., BATES, S.E., MCMANAWAY, M.E. & LIPPMAN, M.E. (1986). Characterisation of estrogen responsive transforming activity in human breast cancer cell lines. *Cancer Res.*, **46**, 1707.

IBBOTSON, K.J., HARROD, J., GOWEN, M. & 5 others (1986). Human recombinant transforming growth factor stimulates bone resorption and inhibits formation *in vitro*. *Proc. Natl Acad. Sci. USA*, **83**, 2228.

MARQUARDT, H., HUNKAPILLER, M.W., HOOD, L.E. & 4 others (1983). Transforming growth factors produced by retrovirus-transformed rodent fibroblasts and human melanoma cells: amino acid sequence homology with epidermal growth factor. *Proc. Natl Acad. Sci. USA*, **80**, 4684.

MASSAGUE, J. (1983). Epidermal growth factor-like transforming growth factor. II. Interaction with epidermal growth factor receptors in human placenta membranes and A431 cells. *J. Biol. Chem.*, **258**, 13614.

MOSES, H.L. (1984). Growth factors and transforming growth factors: potential roles in cancer. *Proc. Am. Assoc. Cancer Res.*, **25**, 412.

MUNSON, P.J. & RODBARD, D. (1980). LIGAND: versatile computerised approach for characterisation of all ligand binding systems. *Anal. Biochem.*, **107**, 220.

PERROTEAU, I., SALOMON, D., DEBORTOLI, M. & 5 others (1986). Immunological detection and quantitation of alpha transforming growth factors in human breast carcinoma cells. *Breast Cancer Res. Treat.*, **7**, 201.

SALOMON, D.S., ZWIEBEL, J.A., BANO, M., LOSANCZY, I., FEHNEL, P. & KIDWELL, W.R. (1984). Presence of transforming growth factors in human breast cancer cells. *Cancer Res.*, **44**, 4067.

SALOMON, D.S., BANO, M. & KIDWELL, W.R. (1986). Polypeptide growth factors and the growth of mammary epithelial cells. In *Breast Cancer: Origins, Detections and Treatment*, Rich, M.A., Hayer, J.C. & Papadimitriou, J.T. (eds) p. 42. Martinus Nijhoff: Boston, MA.

SALOMON, D.S., PERROTEAU, I., RIDWELL, W.R., TAM, J. & DERYNCK, R. (1987). Loss of growth responsiveness to epidermal growth factor and enhanced production of alpha-transforming growth factors in ras-transformed mouse mammary epithelial cells. *J. Cell. Physiol.*, **130**, 397.

SPORN, M.B. & TODARO, G.J. (1980). Autocrine secretion and malignant transformation of cells. *N. Engl. J. Med.*, **303**, 878.

SPORN, M.B. & ROBERTS, A.B. (1985). Autocrine growth factors and cancer. *Nature*, **313**, 745.

STERN, P.H., KRIEGER, N.S., NISSENSON, R.A. & 4 others (1985). Human transforming growth factor-alpha stimulates bone resorption *in vitro*. *J. Clin. Invest.*, **76**, 2016.

STONE, K.R., MICKEY, D.D., WUNDERLI, H., MICKEY, G.H. & PAULSON, D.F. (1978). Isolation of a human prostate carcinoma cell line (DU145). *Int. J. Cancer*, **21**, 274.

STROMBERG, K., HUDGINS, W.R., FRYLING, C.M. & 5 others (1986). Human A673 cells secrete high molecular weight EGF-receptor binding growth factors that appear to be immunologically unrelated to EGF or TGFα. *J. Cell. Biochem.*, **32**, 247.

VALVERIUS, E.M., BATES, S.E., STAMPFER, M.R. & 5 others (1989). Transforming growth factor a production and epidermal growth factor receptor expression in normal and oncogene, transformed human mammary epithelial cells. *Mol. Endocrinol.*, **3**, 203.

VAN ZOELEN, J.J., VAN ROOIJEN, M.A., VAN OOSTWAARD, T.M.J. & DE LAAT, S.W. (1987). Production of transforming growth factors by simian sarcoma virus-transformed cells. *Cancer Res.*, **47**, 1582.

VERBECK, W., BOKEMEYER, C., FALK, H. & SCHMOLL, H.-J. (1988). Growth requirements, growth factor responsiveness and growth factor secretion of three human embryonal carcinoma cell lines. *J. Cancer Res. Clin. Oncol.*, **114**, 553.

WILDING, G., VALVERIUS, E.M., KNABBE, C. & GELMANN, E.P. (1989). Role of transforming growth factor-α in human prostate cancer cell growth. *Prostate*, **15**, 1.

WOLLENBERG, G.K., HARRIS, L., FARBER, E. & HAYES, M.A. (1989). Inverse relationship between epidermal growth factor induced proliferation and expression of high affinity surface epidermal growth factor receptors in rat hepatocytes. *Lab. Invest.*, **60**, 254.

ZWIEBEL, J.A., DAVIES, M.R., KOHN, E., SALOMON, D.S. & KIDWELL, W.R. (1982). Anchorage-independent growth-conferring factor production by rat mammary tumour cells. *Cancer Res.*, **42**, 5117.


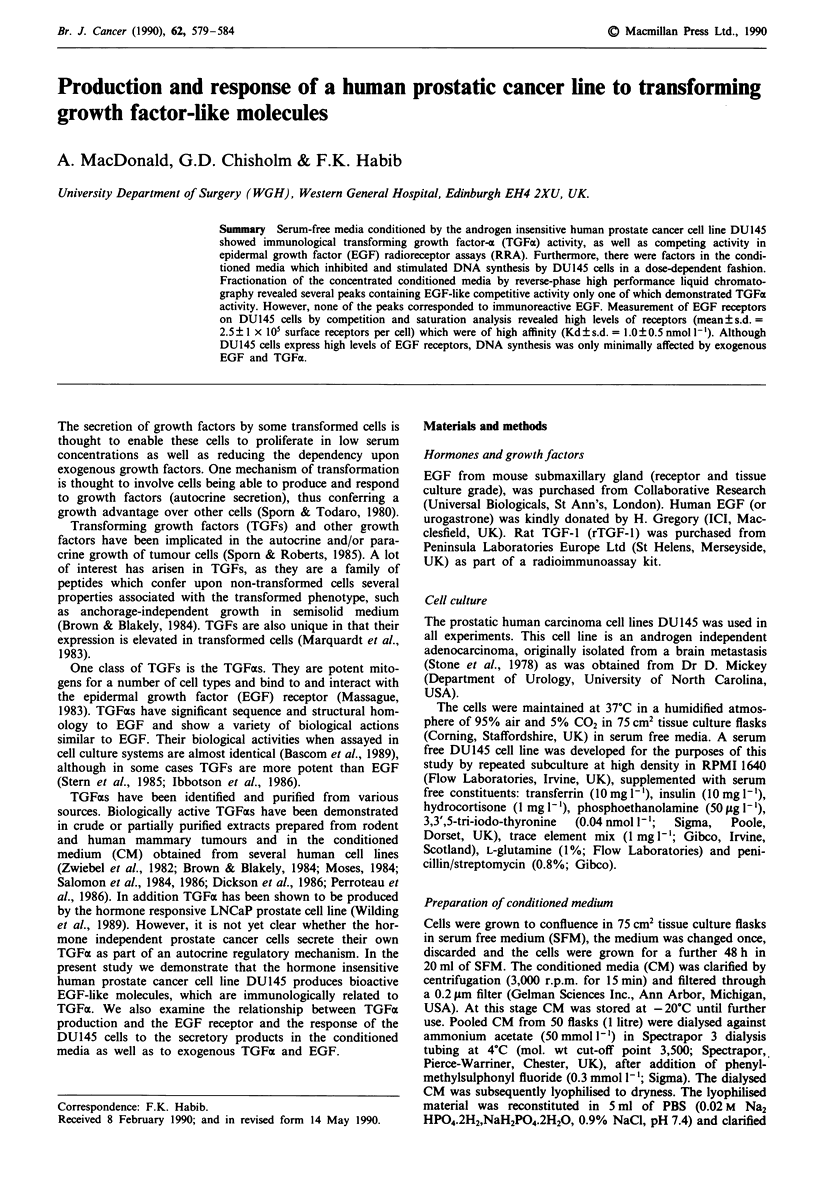

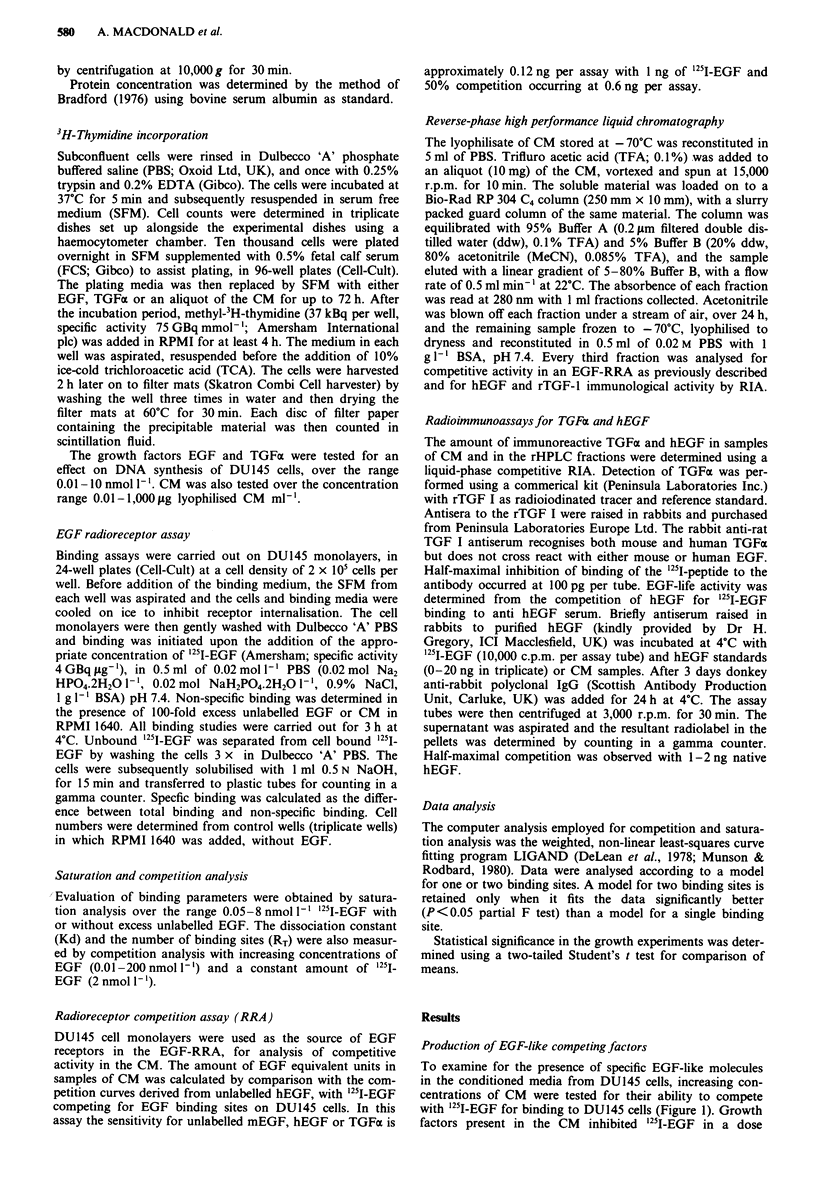

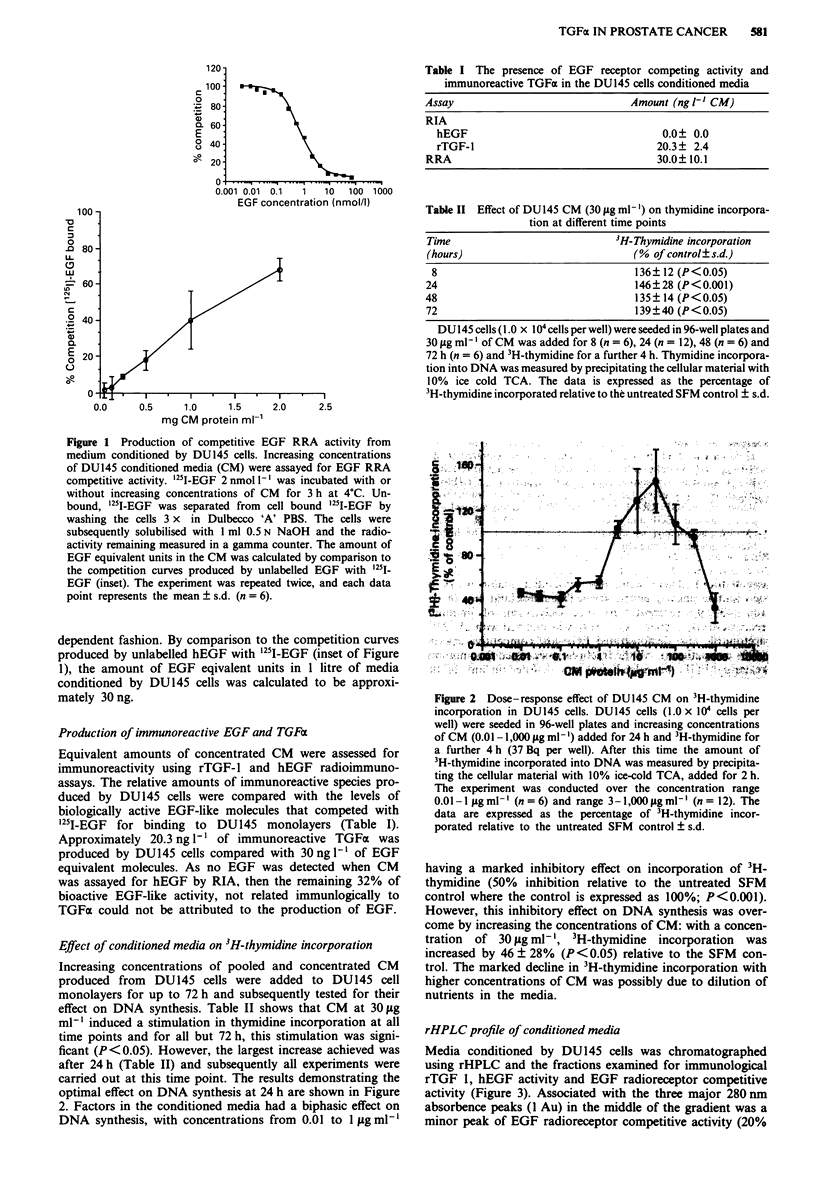

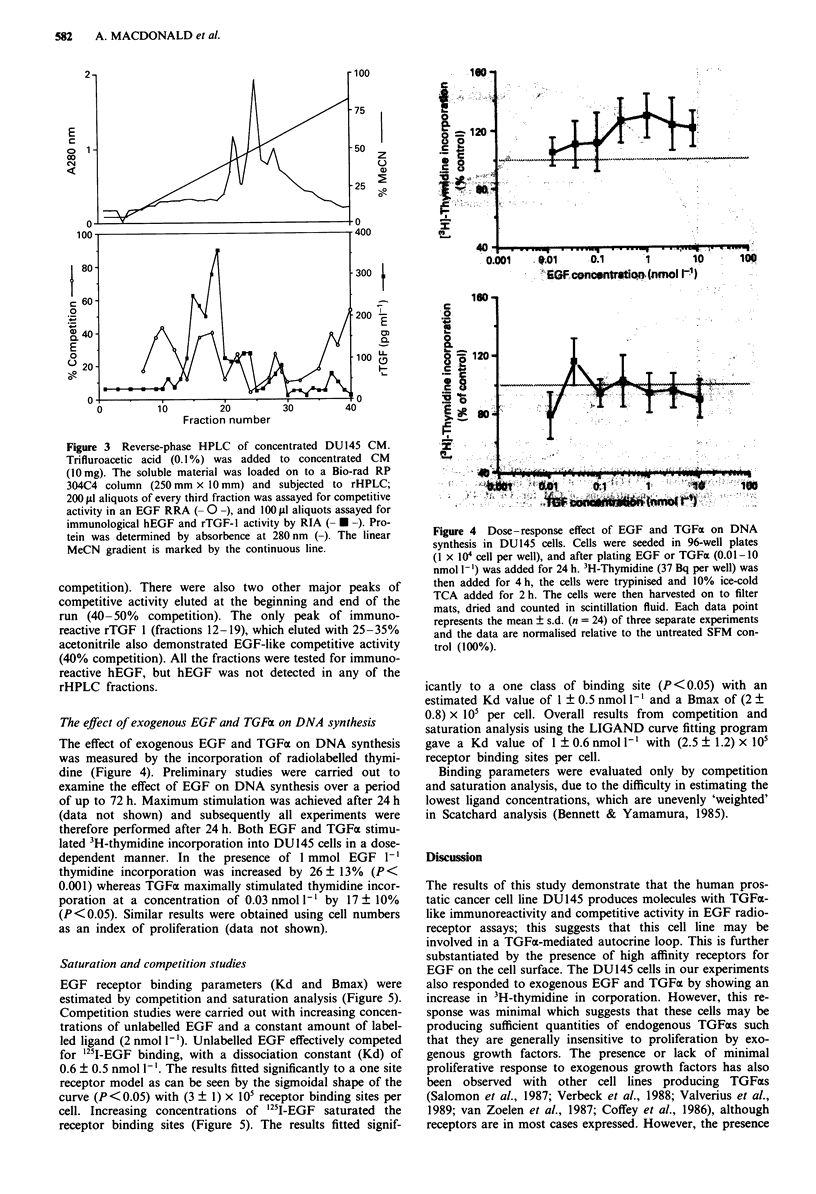

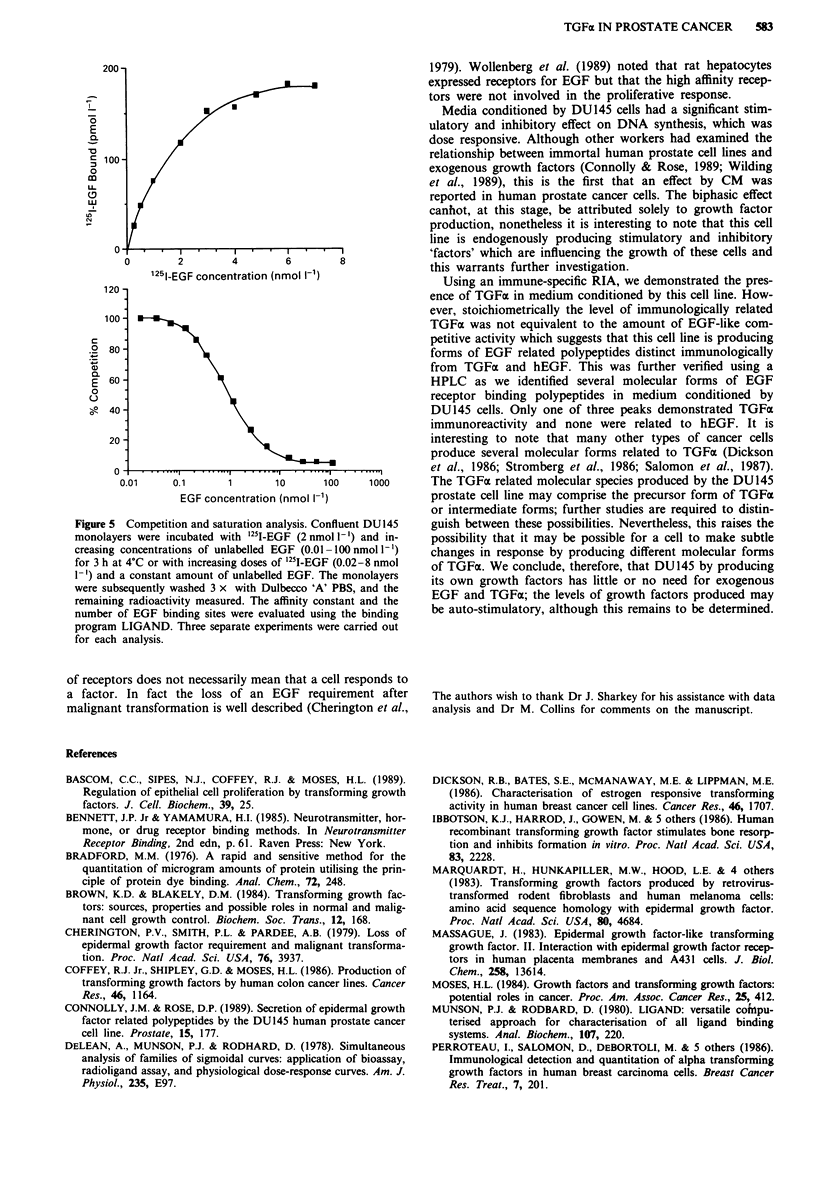

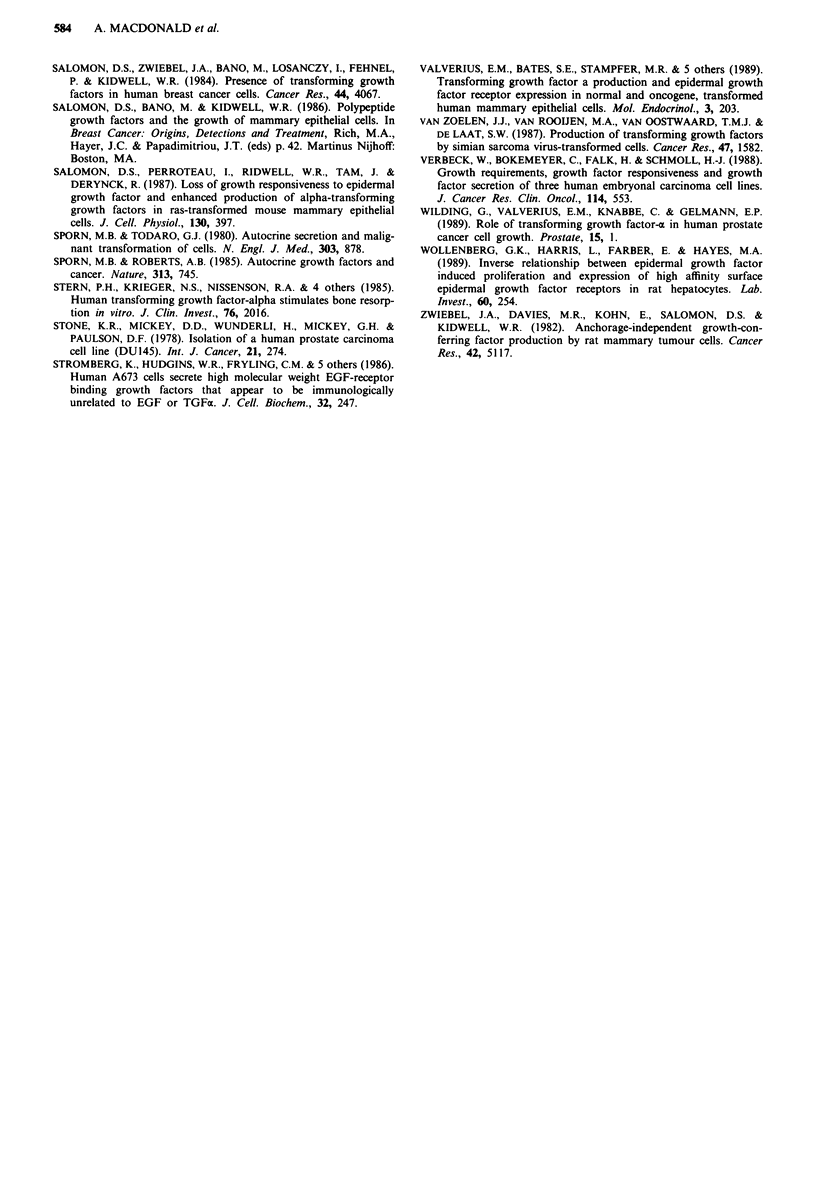


## References

[OCR_00704] Bascom C. C., Sipes N. J., Coffey R. J., Moses H. L. (1989). Regulation of epithelial cell proliferation by transforming growth factors.. J Cell Biochem.

[OCR_00714] Bradford M. M. (1976). A rapid and sensitive method for the quantitation of microgram quantities of protein utilizing the principle of protein-dye binding.. Anal Biochem.

[OCR_00719] Brown K. D., Blakeley D. M. (1984). Transforming growth factors: sources, properties and possible roles in normal and malignant cell growth control.. Biochem Soc Trans.

[OCR_00724] Cherington P. V., Smith B. L., Pardee A. B. (1979). Loss of epidermal growth factor requirement and malignant transformation.. Proc Natl Acad Sci U S A.

[OCR_00729] Coffey R. J., Shipley G. D., Moses H. L. (1986). Production of transforming growth factors by human colon cancer lines.. Cancer Res.

[OCR_00734] Connolly J. M., Rose D. P. (1989). Secretion of epidermal growth factor and related polypeptides by the DU 145 human prostate cancer cell line.. Prostate.

[OCR_00739] DeLean A., Munson P. J., Rodbard D. (1978). Simultaneous analysis of families of sigmoidal curves: application to bioassay, radioligand assay, and physiological dose-response curves.. Am J Physiol.

[OCR_00745] Dickson R. B., Bates S. E., McManaway M. E., Lippman M. E. (1986). Characterization of estrogen responsive transforming activity in human breast cancer cell lines.. Cancer Res.

[OCR_00749] Ibbotson K. J., Harrod J., Gowen M., D'Souza S., Smith D. D., Winkler M. E., Derynck R., Mundy G. R. (1986). Human recombinant transforming growth factor alpha stimulates bone resorption and inhibits formation in vitro.. Proc Natl Acad Sci U S A.

[OCR_00755] Marquardt H., Hunkapiller M. W., Hood L. E., Twardzik D. R., De Larco J. E., Stephenson J. R., Todaro G. J. (1983). Transforming growth factors produced by retrovirus-transformed rodent fibroblasts and human melanoma cells: amino acid sequence homology with epidermal growth factor.. Proc Natl Acad Sci U S A.

[OCR_00762] Massagué J. (1983). Epidermal growth factor-like transforming growth factor. II. Interaction with epidermal growth factor receptors in human placenta membranes and A431 cells.. J Biol Chem.

[OCR_00771] Munson P. J., Rodbard D. (1980). Ligand: a versatile computerized approach for characterization of ligand-binding systems.. Anal Biochem.

[OCR_00776] Perroteau I., Salomon D., DeBortoli M., Kidwell W., Hazarika P., Pardue R., Dedman J., Tam J. (1986). Immunological detection and quantitation of alpha transforming growth factors in human breast carcinoma cells.. Breast Cancer Res Treat.

[OCR_00796] Salomon D. S., Perroteau I., Kidwell W. R., Tam J., Derynck R. (1987). Loss of growth responsiveness to epidermal growth factor and enhanced production of alpha-transforming growth factors in ras-transformed mouse mammary epithelial cells.. J Cell Physiol.

[OCR_00807] Sporn M. B., Roberts A. B. Autocrine growth factors and cancer.. Nature.

[OCR_00803] Sporn M. B., Todaro G. J. (1980). Autocrine secretion and malignant transformation of cells.. N Engl J Med.

[OCR_00811] Stern P. H., Krieger N. S., Nissenson R. A., Williams R. D., Winkler M. E., Derynck R., Strewler G. J. (1985). Human transforming growth factor-alpha stimulates bone resorption in vitro.. J Clin Invest.

[OCR_00816] Stone K. R., Mickey D. D., Wunderli H., Mickey G. H., Paulson D. F. (1978). Isolation of a human prostate carcinoma cell line (DU 145).. Int J Cancer.

[OCR_00821] Stromberg K., Hudgins W. R., Fryling C. M., Hazarika P., Dedman J. R., Pardue R. L., Hargreaves W. R., Orth D. N. (1986). Human A673 cells secrete high molecular weight EGF-receptor binding growth factors that appear to be immunologically unrelated to EGF or TGF-alpha.. J Cell Biochem.

[OCR_00827] Valverius E. M., Bates S. E., Stampfer M. R., Clark R., McCormick F., Salomon D. S., Lippman M. E., Dickson R. B. (1989). Transforming growth factor alpha production and epidermal growth factor receptor expression in normal and oncogene transformed human mammary epithelial cells.. Mol Endocrinol.

[OCR_00837] Verbeek W., Bokemeyer C., Falk H., Schmoll H. J. (1988). Growth requirements, growth factor responsiveness, and growth factor secretion of three human embryonal carcinoma cell lines.. J Cancer Res Clin Oncol.

[OCR_00843] Wilding G., Valverius E., Knabbe C., Gelmann E. P. (1989). Role of transforming growth factor-alpha in human prostate cancer cell growth.. Prostate.

[OCR_00848] Wollenberg G. K., Harris L., Farber E., Hayes M. A. (1989). Inverse relationship between epidermal growth factor induced proliferation and expression of high affinity surface epidermal growth factor receptors in rat hepatocytes.. Lab Invest.

[OCR_00855] Zwiebel J. A., Davis M. R., Kohn E., Salomon D. S., Kidwell W. R. (1982). Anchorage-independent growth-conferring factor production by rat mammary tumor cells.. Cancer Res.

[OCR_00833] van Zoelen E. J., van Rooijen M. A., van Oostwaard T. M., de Laat S. W. (1987). Production of transforming growth factors by simian sarcoma virus-transformed cells.. Cancer Res.

